# The Effect of Confidence Rating on a Primary Visual Task

**DOI:** 10.3389/fpsyg.2019.02674

**Published:** 2019-11-27

**Authors:** Taly Bonder, Daniel Gopher

**Affiliations:** Faculty of Industrial Engineering and Management, Technion – Israel Institute of Technology, Haifa, Israel

**Keywords:** confidence rating, executive control, visual acuity, task formation, spatial attention, reactivity, metacognition

## Abstract

The current study explored the influence of confidence rating on visual acuity. We used brief exposures of the Landolt gap discrimination task, probing the primary visual ability to detect contrast. During 200 practice trials, participants in the Confidence Rating group rated their response-confidence in each trial. A second (Time Delay) group received a short break at the end of each trial, equivalent to the average rating response time of the Confidence Rating group. The third (Standard Task) group performed the Landolt gap task in its original form. During practice, the Confidence Rating group developed an efficient monitoring ability indicated by a significant correlation between accuracy and confidence rating and a moderate calibration index score. Following practice, all groups performed 400 identical test trials of the standard Landolt gap task. In the test trials, the Confidence Rating group responded more accurately than the control groups, though it did not differ from them in response time for correct answers. Remarkably, the Confidence Rating group was significantly slower when making errors, compared the control groups. An interaction in learning efficiency occurred: the Confidence Rating group significantly improved its reaction times after the initial practice, as compared to both control groups. The findings demonstrate an effect of confidence rating on the formation of processing and response strategies, which granted participants significant benefits in later performance.

## Introduction

Do people perceive basic visual input differently, depending on their experience? Some studies imply that the perception of low-level visual information is affected by prior knowledge and is susceptible to priming (see Vetter and Newen, 2014, for a review). In this study, we question whether one can endogenously affect the perception of fine details. We use a version of a Landolt gap task, in which observers detect the location of a small gap, shown on a black-line square. This task was developed by optometrists to examine visual acuity, testing resolution - the ability to detect contrast between the black target and the white gap (Westheimer, 2017). It is a low-level perceptual task, in which performance ceiling can be reached within a small number of trials (Westheimer, 2001). Due to its simplicity, the Landolt gap task became customary in research of visual attention. In this field, the square target is commonly presented peripherally and for a very short period of time – making the observers susceptible to manipulations of visual-spatial attention (Carrasco, 2011). In some cases, the target is displayed in varying peripheral locations, with or without cues that sign its future positioning.

For example, Yeshurun and Carrasco (1999) used a cued Landolt gap task to examine whether spatial attention can improve visual acuity. The authors used peripheral cues to trigger exogenous attention – a pre-saccadic, involuntary orienting response (Jonides, 1981). By allocating observers’ attention toward the square target, the authors showed a significant improvement in visual acuity, manifested in increased response accuracy and a decreased reaction time for gap discrimination. As the cues triggered exogenous response, attentional benefit caused by them was involuntary, implying enhancement of performance by automatic attention orienting. Later, Montagna et al. (2009) extended this finding by revealing that the benefit in visual acuity at attended locations is complimented by an equivalent cost at unattended locations. The authors manipulated exogenous attention by uninformative cues. The cues were either valid, preceded the target in its future location; invalid, appeared in a distracting location; or neutral, appeared at both valid and invalid locations. Even in this relatively complex cued form of the Landolt gap task, the learning asymptote was assumed to be reached within initial 200 trials (Fiorentini and Berardi, 1981). Accordingly, both Yeshurun and Carrasco (1999) and Montagna et al. (2009) reported the effects of exogenous attention only on post-practice performance.

Spatial visual attention has been broadly discussed within the framework of three attention systems (Posner and Petersen, 1990). According to this framework, the attentional system encompasses three distinct functional networks: alerting - attentional preparation for the incoming events; orienting – aligning attention with the physical source of the incoming signal; and executive control – attentional ability to engage in planning, detect errors and keep thought “on task”, to achieve the selected goals. Alerting and orienting are involuntary, automatic processes. Supplementing them, executive control manifests high cognitive involvement. Recently, Bonder et al. (2018) examined whether involuntary visual attention in the cued Landolt gap task can be enhanced by external stimulation of the visual cortex. They applied transcranial Direct Current Stimulation (tDCS) for 15 min of initial practice, and found that in post-stimulation trials tDCS group showed significantly stronger attention orienting, compared to sham control. Reaction times were lower in valid cued trials (attentional benefit) and higher in invalid trials (attentional cost) compared to neutrally cued trials - an effect which was significantly stronger in the tDCS group. The increase in attentional cost and benefit in the tDCS group was of similar magnitude, suggesting that tDCS applied during task formation influenced the overall process of attention orienting. Different task formats shaped during the initial practice in tDCS and sham groups, affected automatic responses only. The results did not imply any effect of executive control on performance, and consistent with literature on exogenous attention, the authors did not test for executive control effects.

Executive control research typically focuses on high-level functions, examining processes broader than early vision (e.g., Baddeley and Della Sala, 1996;Perner and Lang, 1999; Rubinstein et al., 2001). One of such high processes is metacognition, the ability to evaluate the stream of thought (Nelson, 1996). Monitoring of cognitive processes and exertion of control, based on the product of monitoring, are the two pillars of metacognition (Nelson and Narens, 1994). Typically, monitoring abilities correlate with performance – forming an effect by which “we don’t know what we don’t know”, and on the other hand, execute efficient metacognition with expertise (Dunning, 2011). Metacognitive ability is measured by comparing the objective performance with subjective evaluation of that performance. For example, in word-pair memory tasks participants commonly report Judgment of Learning – the subjective confidence to recall each learnt pair. In perceptual experiments, confidence in accuracy of the perceptual report, termed confidence rating, is collected (Fleming and Lau, 2014).

Assessment of metacognitive measures has been shown to cause reactivity – a change in performance resulting from the metacognitive evaluation (Rhodes and Tauber, 2011). For example, Double and Birney (2017) studied whether confidence rating can influence performance in Raven’s Progressive Matrices test. They found that delivering confidence rating after each experimental trial significantly improved task performance, constructing positive reactivity. However, the underlying mechanisms of the reactivity effect remain unclear, and reactivity effects on early perception are yet to be studied (Double et al., 2018). Metacognitive reactivity effect is congruent with the literature on task formation, according to which targeted variations in task demands result in adoption of dissimilar cognitive strategies, yielding differences in following performance. Previous research shows that manipulations applied during early experience with a task, the stage at which processing and response formats were developed, significantly influence post-practice behavior (e.g., Gopher, 1984; Gopher et al., 1985, 2000; Seagull and Gopher, 1997). Accordingly, Gopher et al. (1989) showed that specific task demands shape participants’ cognitive strategies to obtain the task goal. Arguably, the process of task formation integrates task properties into a combined internal representation, thus enabling formalization of goal-directed strategies, tailored to that representation. Bonder et al. (2018) have demonstrated the effect of task formation in the cued Landolt gap task where experimental manipulation exerted in practice caused prominent results in test.

Relying on the findings of Gopher et al. (1989), Double and Birney (2017), and Bonder et al. (2018), in the current study we aimed to inspect how confidence rating, introduced during the initial encounter with the task, will affect the post-practice performance. We hypothesized that integrating confidence rating even in a primary visual task such as the cued Landolt gap, will construct an internally enriched task formation, amplifying the effects of monitoring and control, and influencing performance as participants gain experience with the task. In the stage of task formation, we predicted a positive correlation between confidence rating and response accuracy, according to the literature on metacognition (Adams and Adams, 1961; Vickers, 1979). Finally, we aimed to explore whether any indicators of improvement in goal-directed behavior will be found in post-practice trials, when confidence rating is not requested anymore.

## Materials and Methods

**FIGURE 1:**
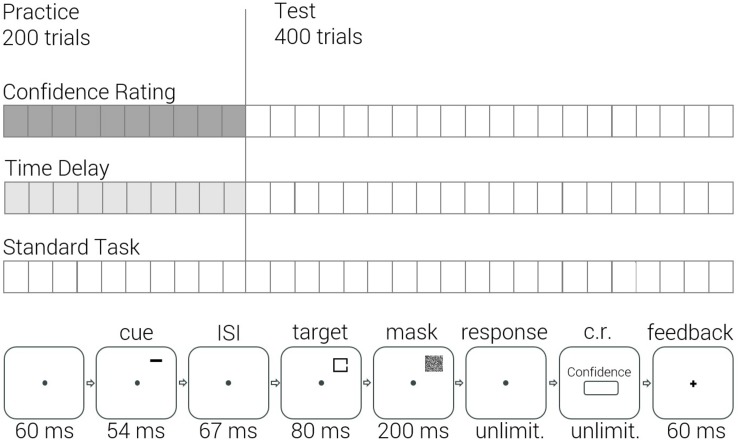
Experimental design **(top)** and a single trial design of the Confidence Rating group in practice **(bottom)**.

A modified version of the task employed by Yeshurun and Carrasco (1999) was used. Three groups participated in the study and performed the cued Landolt gap task. During the practice stage, participants of the Confidence Rating group rated their confidence after each gap discrimination. Similarly, Time Delay control group participants received time intervals after each gap discrimination. These intervals were applied only during the practice stage and were equivalent to the average confidence rating response time of the Confidence Rating group. Finally, Standard Task group performed the cued task without any additional manipulations. Following the practice, in the test stage, all groups performed the cued Landolt gap task under identical standard conditions, i.e., no groups reported confidence, Figure 1. Results of the Standard Task group have been reported in Bonder et al. (2018), this baseline group performance enabled to methodologically compare between the two lines of research.

### Participants

Forty-four undergraduates from University of Haifa subject pool (*M*_age_ = 25.2, 27 female) participated in the experiment, all with normal or corrected to normal vision, right handed, and naive to the purpose of the study. Standard Task group consisted of 14 participants, while Confidence Rating and Time Delay groups consisted of 15 participants each. All participants gave written informed consent according to the declaration of Helsinki guidelines, as approved by the institutional ethics committee of University of Haifa (307/15). All participants received 30 NIS (8.30$) for participation.

### Apparatus

Participants viewed the stimuli on a 21 inch CRT color monitor, in a resolution of 1,024 × 768 pixels, with a refresh rate of 60 Hz. They viewed the display binocularly from a distance of 57 cm. and responded by pressing a key on a keyboard with the index or the middle finger of their right hand. The task was generated by E-prime on Windows powered computer.

### Procedure

A square with a gap at one of its sides was presented in one of two possible locations. Observers were asked to indicate which side of the square contained the gap. A valid, invalid or neutral pre-cue appeared before the square, and was used to draw observers’ attention to a specific location. The cue was exogenous and was presented for a short period of time so that eye movements would not take place. Observers read instructions specifying the target, advising them to focus on a central fixation point throughout the experiment, and asking them to indicate, as rapidly and accurately as possible, whether a gap is on the left or right side of the square. In each trial, accuracy and response time from target onset were registered. At the end of each trial, a plus or a minus sign served as feedback. Experiment duration was nearly 1 h, depending on individual response times.

The experimental session contained a total of 600 trials – 10 blocks of 20 trials in practice and 20 blocks of 20 trials in test. Square location, gap side and pre-cue types were randomized between the trials. Each block was followed by a report of accuracy ratio achieved in it, and then by a short break which was terminated by the observer. In each trial of the practice stage, participants of the Confidence Rating group rated their confidence in the provided answer. Using a keyboard, they entered a number between 0 (a complete guess) and 100 (I’m absolutely sure). The confidence rating stage was then followed by a feedback sign, Figure 1. The participants of the Time Delay group received a time interval of 1500 msec. between the response and the feedback, matching the average time it took the Confidence Rating group to rate confidence in a single trial. This waiting period was added to the practice stage only. In test, all groups performed the same standard task.

### Stimuli

A black-line square appeared on a white background, in one of two possible locations – the upper or the lower right side of the visual field (in consistency with the method reported in Bonder et al., 2018), at eccentricity of 6° from the center of the display. The square subtended 1 × 1° of visual angle and contained a gap of 0.1° in one of its sides – left or right, with equal probability. The square appeared for 80 ms to keep overall performance level at 75–85% correct, so that ceiling or floor effects would be avoided. A 1.4 × 1.4° square of distorted lines served as a visual mask and was presented after the square’s disappearance, at its location, for 200 ms. Thus, eye movements could not take place while the display was present, since about 250 ms are needed for saccades to occur (Mayfrank et al., 1987). A black fixation dot (0.15° diameter) was presented in the center of the screen throughout the experiment. At the end of each trial, a plus (0.33° height 0.33° width) or a minus (0.33° width 0.14° height) black sign served as feedback and was presented at the center of the display for 1000 ms.

Prior to the target appearance, an exogenous pre-cue was used to draw observers’ attention to a certain location on the display. The pre-cue appeared for 54 ms. and after an ISI of 67 ms (i.e., a SOA of 121 ms.) the square target was presented. To prevent spatial masking effects, the pre-cue appeared 0.3° above the location of the target. The pre-cue was a green (0, 128, 0 in standard RGB color space) horizontal bar, subtending 0.5° width 0.14° height of visual angle. In one third of trials the pre-cue was valid – it indicated the location in which the square will be presented. In one third of trials, it was invalid – the horizontal bar appeared above a location in which the square was not presented. In one third of the trials, it was neutral - two identical bars appeared above the two possible locations in one of which the square could be presented later. The bars indicated that the square had an equal probability of appearing at each location.

## Results

We first inspected the stage of task formation in the Confidence Rating group, and then tested whether this formation affected performance relatively to the two control groups. Lastly, we examined whether local executive control influence was evident in the Confidence Rating group relatively to the control groups.

### Task Formation of the Confidence Rating Group

**FIGURE 2:**
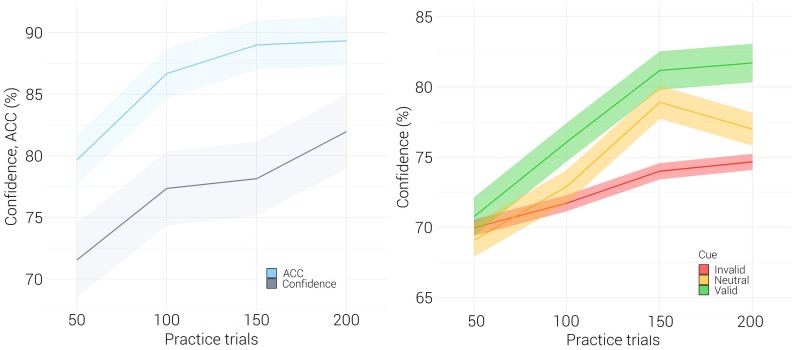
Mean accuracy and mean confidence rating of the Confidence Rating group in practice. **Left** - confidence rating (gray) and accuracy (blue). **Right** - confidence rating in Valid (green), Neutral (yellow) and Invalid (red) conditions. Standard error is shown by a ribbon.

To inspect the task formation of the Confidence Rating group, accuracy and confidence rating in practice were examined. We calculated calibration, the goodness of fit between the proportion of correct responses and confidence rating, using the calibration index:$\frac{1}{N}\,\sum_{i=1}^{N}{\left(c_{i}-p_{i}\right)}^{2}$; and resolution – the extent to which correct and incorrect answers are assigned to different confidence rating categories, using the resolution index: $\frac{1}{N}\,\sum_{i=1}^{N_{c}}\left(c_{i}\,c\,o\,r\,r\,e\,c\,t\right)-\sum_{i=1}^{N_{i}}\left(c_{i}\,i\,n\,c\,o\,r\,r\,e\,c\,t\right)$ (Nelson, 1984; Schraw, 2009). The group displayed a general trend of under-confidence (*C* = −8.84) and moderate resolution (*G* = 0.60), Figure 2 left. Bootstrap analyses performed on Spearman’s rho scores revealed a strong positive correlation between accuracy and confidence (*r*_*s(*__15__)_ = 0.74, *p* < 0.001). This correlation was significant in valid (*r*_*s(*__15__)_ = 0.77, *p* < 0.001), invalid (*r*_*s(*__15__)_ = 0.72, *p* = 0.003) and neutral (*r*_*s(*__15__)_ = 0.54 *p* = 0.04) cue conditions.

To test whether accuracy and confidence rating measures were sensitive to cue manipulation, two analyses of variance (ANOVA) were performed. Repeated measures ANOVA (cue: Valid, Invalid, Neutral) revealed that accuracy of the Confidence Rating group in practice was sensitive to the exogenous cue [*F*_(__2,28__)_ = 5.57, *p* = 0.009, η^2^ = 0.29]. Bonferroni correction for multiple comparisons showed that the effect stemmed from a significant difference between valid (Mean = 0.89, SD = 0.06) and invalid (Mean = 0.84, SD = 0.07) cue conditions (*p* = 0.008). Repeated measures ANOVA performed on confidence rating (cue: Valid, Invalid, Neutral; accuracy: Correct, Error) showed that confidence rating was marginally significantly sensitive to the effect of cue [*F*_(__2,28__)_ = 3.33, *p* = 0.051, η^2^ = 0.19]. Similarly to the effect obtained in accuracy, a difference between valid (Mean = 69.93, SD = 6.67) and invalid (Mean = 64.24, SD = 6.03) cue conditions (*p* = 0.034) emerged. The outcomes demonstrate that participants could monitor the attentional benefit in accuracy caused by the valid cues and the cost caused by the invalid cues, Figure 2 right.

### Comparative Performance in Practice and in Test

To inspect the influence of confidence rating on the establishment of a stable task format, we tested for differences in accuracy and in response time between the groups, both in practice and test phases. Mixed effects ANOVA (group: Confidence Rating, Time Delay, Standard Task; phase: Practice, Test; cue: Valid, Neutral, Invalid) were performed. Accuracy was tested using average individual performance. Average individual response time for correct answers within ±2SD range (20135 trials) and average individual response time for error answers within the ±2SD range (4507 trials) were analyzed separately. Following the tests, Bonferroni corrections for multiple comparisons were performed. To compare the initial performance levels of the three groups, one-way ANOVA was conducted on accuracy in the first block of practice, 20 trials [*F*_(__2,41__)_ = 0.52 *p* = 0.6]. Full data can be found at DOI: 10.17605/OSF.IO/M96T.

Analysis of accuracy revealed the main effects of group [*F*_(__2,41__)_ = 3.68, *p* = 0.034, η^2^ = 0.15], experimental phase [*F*_(__1,41__)_ = 4.12, *p* = 0.049, η^2^ = 0.09] and exogenous cue [*F*_(__2,82__)_ = 7.11, *p* = 0.001, η^2^ = 0.15]. No significant interactions occurred (all *p*’s > 0.35). As expected, the groups improved their accuracy with practice. The exogenous cue affected performance: Bonferroni pairwise comparisons revealed the difference between valid (Mean = 0.83, SD = 0.14) and invalid (Mean = 0.81, SD = 0.13) cued trials (*p* = 0.002). Throughout the experiment, the Confidence Rating group performed significantly more accurately than the Standard Task group (*p* = 0.044), but did not differ significantly from the Time Delay group (*p* = 0.089). Inspecting the group performance in test, one-way ANOVA [*F*_(__2,41__)_ = 4.38, *p* = 0.19] revealed that the Confidence Rating group responded significantly more accurately than the Standard Task group (*p* = 0.036) and marginally significantly more accurately than the Time Delay group (*p* = 0.053).

Correct response time analysis yielded three significant main effects: group [*F*_(__2,41__)_ = 5.97, *p* = 0.005, η^2^ = 0.23], phase [*F*_(__1,41__)_ = 42.27, *p* < 0.001, η^2^ = 0.51] and cue [*F*_(__1_._93,79_._05__)_ = 9.31, *p* < 0.001, η^2^ = 0.19]. As hypothesized, the groups reduced latency with learning (*p* < 0.001) and the effect of exogenous cue differentiated between valid (Mean = 597.42, SD = 148.3) and invalid (Mean = 624.98, SD = 160.88) cued trials (*p* = 0.004). A significant interaction between group and experimental phase occurred [*F*_(__2,41__)_ = 8.41, *p* = 0.001, η^2^ = 0.29]. In practice, the Confidence Rating group responded significantly slower than the Time Delay group (*p* = 0.002) and marginally slower than the Standard Task group (*p* = 0.081), but no such difference was found in test (all *p*’s > 0.15). The prolonged latency of the Confidence Rating group in practice can be explained by a higher processing demand, as will be inspected in discussion. The results show that during test, the benefit of the Confidence Rating group in accuracy was not accompanied by a cost in response time, implying no speed-accuracy tradeoff. All remaining interactions were non-significant (all *p*’s > 0.13).

Error Response Time reduced significantly from practice to test [phase: *F*_(__1,41__)_ = 33.98, *p* < 0.001, η ^2^ = 0.45], but was not affected by the exogenous cue [*F*_(__2,82__)_ = 0.71, *p* = 0.49]. A significant difference between the groups was found (*F*_(__2,41__)_ = 18.84, *p* < 0.001, η^2^ = 0.48) showing that Confidence Rating group responded significantly slower than the control groups (*p* < 0.001, *p* < 0.001). A significant interaction between phase and group [*F*_(__2,41__)_ = 14.51, *p* < 0.001, η^2^ = 0.42] occurred. One-way analysis of variance (group: Confidence Rating, Time Delay, Standard Task) performed on within subject response-time-differences between the practice and the test phases [*F*_(__2,41__)_ = 14.45, *p* < 0.001] revealed that the Confidence Rating group reduced its response time between the experimental phases significantly more compared to the Time Delay group (*p* < 0.001) and the Standard Task group (*p* < 0.001). However, it remained the slowest group during the test phase.

**FIGURE 3:**
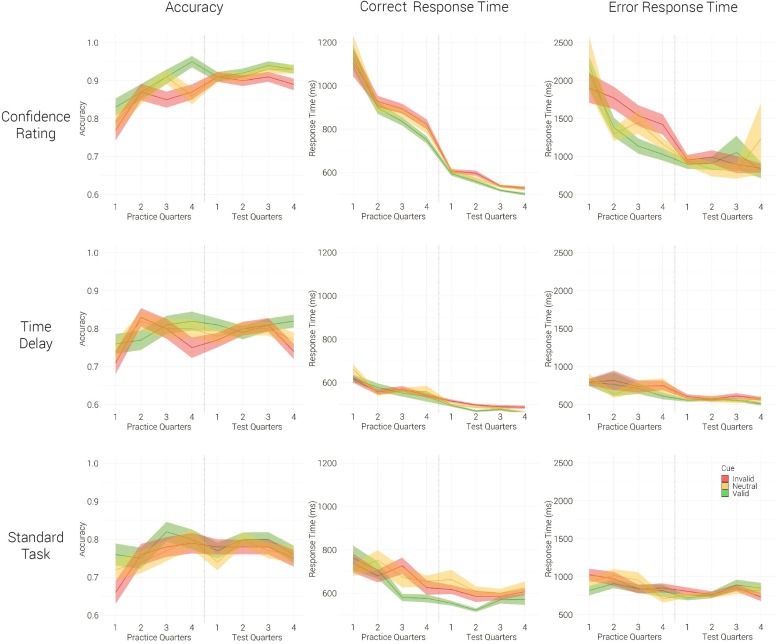
Mean group performance in Valid (green), Neutral (yellow) and Invalid (red) conditions; in accuracy **(left)**, correct response time **(middle)** and error response time **(right)**; in practice and in test. Each practice quarter comprises 50 trials, while each test quarter includes 100 trials. Standard error is shown by a ribbon.

The Confidence Rating group showed a significant advantage over the control groups in test – it responded more accurately and showed no difference in response time for correct answers, Figure 3 and Appendix A. Remarkably, this group produced slower responses compared to the control groups in error trials. Moreover, the group expressed an advantage in learning. This finding was enlightening, as some theoretical assumptions imply that no further learning should take place in the Landolt gap task after the initial 200 trials (Westheimer, 2001); on the other hand, Bonder et al. (2018) demonstrated an improvement in performance afterward. We questioned whether the Confidence Rating group would show improved learning throughout the experiment, capitalizing on the processing strategies formulated during task formation. Both measures of response time yielded significant interactions between group and phase, highlighting the prolonged learning phase of the Confidence Rating group after the initial practice.

### Indicators of Executive Control

We have shown the efficient monitoring ability of the Confidence Rating group in practice and demonstrated its performance benefits after practice. However, whether this group executed better control over the perceptual process, compared to the control groups, remained to be examined. We hypothesized that the Confidence Rating group will benefit from better resolution, an ability that this group acquired during task formation. In test, this benefit will be expressed by an improved ability to subjectively discriminate between the easy (correct response) and more difficult (erroneous response) perceptual trials. If Confidence Rating group could purposely control the processing and response duration, this group would show prolonged responses in difficult trials, compared to the control groups.

**FIGURE 4:**
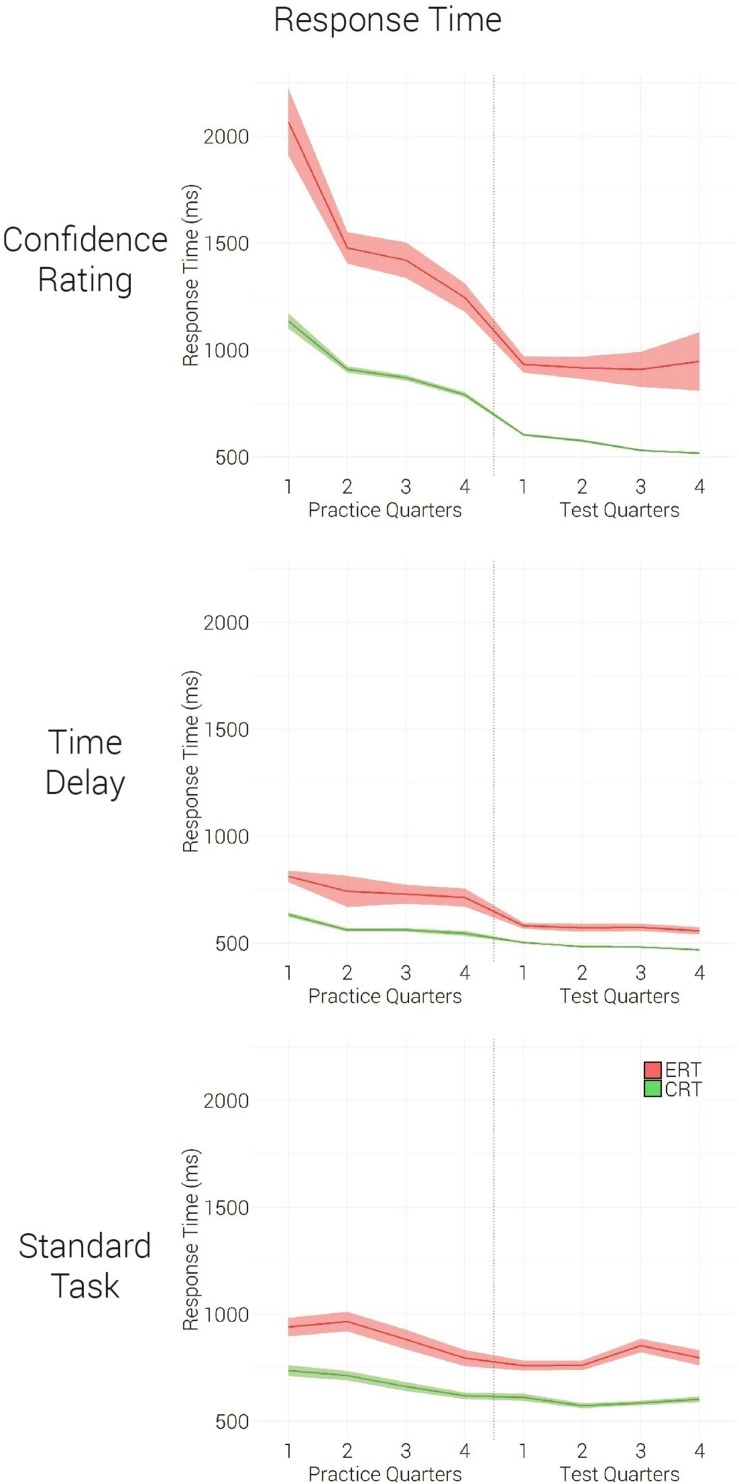
Mean group response time in practice and test quarters, in correct (green) and error (red) trials. Each practice quarter comprises 50 trials, while each test quarter includes 100 trials. Standard error is shown by a ribbon.

For this aim, mixed effects ANOVA (group: Confidence Rating, Time Delay, Standard Task; phase: Practice, Test; accuracy: Correct, Error) was conducted on response times. Three significant main effects [group: *F*_(__2,41__)_ = 14.3, *p* < 0.001, η^2^ = 0.41; phase: *F*_(__1,41__)_ = 43.86, *p* < 0.001, η^2^ = 0.52; accuracy: *F*_(__1,41__)_ = 78, *p* < 0.001, η^2^ = 0.66], three two-way interactions [group-phase: *F*_(__2,41__)_ = 15.09, *p* < 0.001, η^2^ = 0.42; group-accuracy: *F*_(__1,41__)_ = 14.13, *p* < 0.001, η^2^ = 0.41; phase-accuracy: *F*_(__2,41__)_ = 4.99, *p* = 0.31, η^2^ = 0.11] and a three way interaction [group-phase-accuracy: *F*_(__2,41__)_ = 6.79, *p* = 0.003, η^2^ = 0.25] were found, Figure 4. One-way analysis of variance (group: Confidence Rating, Time Delay, Standard Task) performed on within subject response-time-differences between the correct and the error trials revealed that the Confidence Rating group showed a larger response time difference between the correct and the error trials [*F*_(__2,41__)_ = 14.52, *p* < 0.001], compared to the control groups (*p* < 0.001, *p* < 0.001). This effect was stronger during the practice, compared to the test phase (*p* = 0.03). The findings support the hypothesis by which Confidence Rating group acquired an improved ability to monitor trial difficulty and to prolong its processing time.

## Discussion

This study examined whether metacognitive confidence rating can influence performance of a primary visual acuity task. The results show that confidence rating assessment during practice significantly improved performance in the test trials, compared to the control groups. This effect was prominent in accuracy and was not a result of a speed accuracy trade-off, as no difference in correct response time occurred between the Confidence Rating and the control groups. An interaction between experimental stage and group signified an enhanced ability of the Confidence Rating group to continue learning after the initial practice. Finally, the Confidence Rating group showed a stronger differentiation between correct and error trials response time, compared to the control groups. We interpret these benefits as effects of confidence rating on task format, developed during practice.

The improvement of the Confidence Rating group in accuracy is congruent with previous studies on metacognition. The positive effect of metacognition-measures assessment, termed positive reactivity, was reported to be caused by Judgment of Learning in mathematical problem solving (Desoete and Roeyers, 2006) reading (Pressley, 2002) learning from visual items (Prins et al., 2006) and more (for a review see Double et al., 2018). Lately, Double and Birney (2017) showed positive reactivity to confidence rating in Raven’s Progressive Matrices. Note that the Landolt gap is a primary task, compared to the paradigm used in these studies. Yet, the reactivity effect remains methodologically controversial, as only the significant findings addressing it are published, and the effect seems to be exceptionally task-specific (Double et al., 2018). Accordingly, some studies report a negative effect of reactivity (e.g., Mitchum et al., 2016). Relying on previous literature, testing for reactivity effects in a new task is a puzzling experience, as one has no substantial information to predict whether the effect will be positive, negative, or whether it will occur at all. In the current study, we reason that the effect of reactivity, whether positive or negative, is an inevitable result of metacognitive judgment process established during the phase of task formation. As the requirement for metacognitive monitoring is weaved into the task demands, it initiates further cognitive processing, alters task constraints, and requires new strategies for allocation of effort. Reasonably, one could deduce that this kind of task formation efforts would lead to unique subsequent performance. We suggest the prism of task formation stage for examination of previous findings. For example, in a study conducted by Fleming et al. (2012) assessment of confidence rating did not affect performance in a perceptual task. In the task, participants viewed either a face or a house with an embedded visual noise and reported the type of the stimulus in each trial. First, they performed 200 trials of face/house discrimination. Next, in the first ten test trials (block 1), they rated their confidence after each report, and in the next five trials (block 2) performed the perceptual task without confidence rating. Throughout the 75 test trials, participants performed these two blocks in a sequence. The obtained results revealed no difference in accuracy between the two types of test blocks. In light of the current findings, the results could be interpreted in terms of task formation. Arguably, participants gradually combined the task demands into a single representation unit – utilizing this unit throughout the experiment.

To our knowledge, the current study is the first to report an effect of confidence rating on early visual perception. In the current study, the cued Landolt gap task was specifically constructed to enable exogenous attention effects on performance, while limiting the influence of higher-level cognitive processes (Yeshurun and Carrasco, 1999). To recap, the target (80 ms) and the cue (54 ms) were presented for very short durations, well below documented times of voluntary eye movement toward their location. Additionally, the target was immediately masked by a visual noise, to erase its sensory memory, supposedly preventing participants from reflecting on it. Moreover, the exogenous cues were uninformative since they did not bear any predictive information regarding the gap location in the square line. Lastly, the cues were peripheral, automatically driving spatial attention toward the location in which they were presented. In other words, the task was structured to limit voluntary intervention. The exogenous task was used with an embedded requirement to rate confidence, a manipulation designed to induce high-level executive control. The results show that rating of confidence during task formation caused a significant improvement in performance in the following test trials. This effect of task formation in practice is congruent with the results reported by Bonder et al. (2018). There, tDCS applied to the visual cortex only during practice, amplified the effects of attention orienting in the following test trials. Arguably, neuroenhancement of the visual area affected not only the representation of the square target, but also visibility, and thus influence, of the spatial cue. This way, participants of the tDCS group learned the task with emphasis on attention orienting. After stimulation, participants continued using the only task formation they have acquired - emphasized attention orienting to cues. Similarly, we reason that in the current study participants of the Confidence Rating group developed metacognitively guided process monitoring which became an inherent part of the task structure (Gopher et al., 1989; Gopher, 2006). Gap discrimination, response to a spatial cue and confidence rating formed a unified cognitive demand profile. In this, seemingly primary visual discrimination task, participants of the Confidence Rating group integrated the endogenous monitoring effort applied in their ratings. Hence, when established, task formation continued to influence processing throughout the experiment.

Much of the improvement in post-practice performance was achieved in response time for correct answers. Petrusic and Baranski (2003) have previously reported similar results, testing for effects of confidence rating on response time in a perceptual task. In their experiment, participants judged sizes of two squares, rating response confidence after each trial. In this experimental paradigm no presentation time constraints were set – the targets remained visible until a response was obtained. Petrusic and Baranski (2003) found that both response time for size judgment and confidence rating-time diminished with practice. The authors showed that the process of confidence rating occurred both during and after the judgment of square-size. Thus, a proportion of the prolonged responses of the size judgment, found in the Confidence Rating group in their study was explained by the co-occurring confidence rating process. The results of the current study stand in line with these findings and present further evidence of the perseverance of a unified task form, even when the rating of confidence is not required anymore.

In the current study we used the response time for error answers as an explorative measure of executive control. This measure was collected to inspect the results in light of diffusion decision (Ratcliff and McKoon, 2008) and dynamic signal detection (Pleskac and Busemeyer, 2010) models. According to these models, noisy evidence is accumulated until it reaches the response criterion – providing, in our task, either a correct or a wrong answer. This response criterion can be altered by top-down functions, controlling the time needed for evidence accumulation. We found that in the test trials the error responses of the Confidence Rating group took longer than that of both control groups, while no significant difference occurred in correct response times. This finding may imply an improved resolution of the Confidence Rating group, and an improved ability to control behavior, supposedly as a function of response evaluation requirement in each trial. Interestingly, no overall speed accuracy trade-off was found in this group throughout the experience. Early perceptual tasks usually do not involve a speed-accuracy tradeoff, and when examining solely the correct response time, no such tradeoff is evident in the results. During practice, the Confidence Rating group responded significantly slower than control groups, but no significant advantage was found in accuracy; while in the test trials, accuracy of this group was higher but response time for correct answers did not differ between the groups. In this case, inspection of error response time is essential to comprehend the difference in response criterion between the groups. Previous studies show that in tasks which instructions emphasize accuracy over speed – a larger time difference between correct and error responses tends to occur. On the contrary, when fast responses are preferred, the time difference between correct and error trials diminishes (e.g., Pike, 1968; Luce, 1986; Petrusic, 1992). In the current study, all groups received identical instructions, nonetheless confidence rating manipulation seemingly caused participants to emphasize accuracy over latency. This preference emerged at the stage of task formation and persisted throughout the experiment.

During practice, a general trend of under-confidence was observed in the Confidence Rating group. It has long been known that confidence can correlate with objective sensitivity measures of perceptual decisions (Peirce and Jastrow, 1885; Volkmann, 1934). Consistently, confidence is considered to reflect the strength of accumulated sensory evidence (Vickers and Packer, 1982). Contrary to higher-level tasks such as word-memory or mathematical problems in which over-confidence is systematically found (Ehrlinger et al., 2008), in low-level perceptual tasks, under-confidence often emerges (Adams and Adams, 1961; Björkman et al., 1993). In the current study, due to the rapid stimuli appearance, many participants verbally reported that they did not see the cue, and some said that even the target was not always visible to them. The measures of accuracy and confidence increased with learning – low confidence ratings were prevalent in the initial trials while higher scores were obtained with practice. Complementing these results, we found that confidence ratings were marginally susceptible to cue manipulation. Specifically, participants could monitor the attentional costs and benefits caused by orienting of spatial attention. Combining the sensitivity to cueing with the finding by which the Confidence Rating group excelled in post-practice accuracy implies good monitoring abilities. These results are of high importance for feasible implications, for occupations in which observers make decision based on interpretation of visual data. The results suggest that one can reach higher levels of performance, simply shifting the path of the initial practice.

Can assessment of confidence rating result in positive reactivity in a task as low as Landolt gap? Previously, Yeshurun and Carrasco (1999) have shown that deployment of attentional resources can improve visual acuity, explaining this finding by enhancement of target representation. Montagna et al. (2009) attributed the found attentional benefits and costs to cognitive resources limitation – the processing benefit was complimented by an equivalent cost. Both studies proposed a zero-sum framework, in which attention allocates the available cognitive resources. Bonder et al. (2018) used the same task to introduce external neuroenhancement. There, participants had a higher probability to create a vivid representation of the stimuli, due to signal augmentation. The current work proposes an endogenous approach, using executive control intervention to refine visual acuity.

### Limitations and Future Reasearch

The current study focused on the effect of executive control on early perception, therefore confidence rating was used only as a trigger for high-level processes involvement. Since we intended to focus on the effects on performance, rather than on the underlying metacognitive mechanisms – profound metacognitive analyses were not in scope of the current work. Signal detection theory measures were used to confirm that the results in accuracy do not stem from a change in criterion. However, estimation of metacognitive bias, sensitivity and efficiency, as specified by Fleming and Lau (2014), could further clarify the metacognitive characteristics in the development of task formation. Furthermore, the diminishing criterion model (Ackerman, 2014) might shed light on the connection between confidence and response times. Likewise, future studies are needed to research the causal effects of metacognitive measures assessment on the control strategies. The following questions should be addressed: What is needed to fully eliminate executive control, and what is needed to bring its contribution to a maximum, in low-level perceptual tasks? What are the contributions of metacognitive judgments? What are the cognitive and behavioral costs implied by them, and how do they influence the delicate process of task formation?

### Conclusion

Three main conclusions emerge from the current study. Assessment of confidence rating improved performance in a task of early visual perception. This effect was formed during task formation and became evident in the following test trials. The positive effect of confidence rating assessment can be addressed by terms of executive control processes.

## Data Availability Statement

The datasets generated for this study are available online at https://osf.io/m96tj/?view_only=4b3d57ae61af458a831c226d90d1af2b.

## Ethics Statement

The studies involving human participants were reviewed and approved by the Institutional Ethics Committee of University of Haifa (307/15). The patients/participants provided their written informed consent to participate in this study.

## Author Contributions

TB designed the study, acquired, analyzed and interpreted the data, and drafted the manuscript. DG designed the study, analyzed and interpreted the data, and revised the manuscript.

## Conflict of Interest

The authors declare that the research was conducted in the absence of any commercial or financial relationships that could be construed as a potential conflict of interest.

## Appendix A

**Table:** Group performance in invalid, neutral, and valid cue conditions in practice and in test.
